# Real-Time Three-Dimensional Echocardiography and Myocardial Strain: Ready for Use in Clinical Practice

**DOI:** 10.5935/abc.20190179

**Published:** 2019-11

**Authors:** Frederico Jose Neves Mancuso

**Affiliations:** Universidade Federal de São Paulo - Escola Paulista de Medicina - Cardiologia e Medicina de Urgência, São Paulo, SP - Brazil

**Keywords:** Ventricular Function, Left, Echocardiography, Three-Dimensional, Strain, Speckle Tracking, Reference Values

The ability of echocardiography to quantify volumes and to evaluate the contractile function of cardiac chambers has evolved greatly in recent years, particularly due to the development of real-time three-dimensional echocardiography (echo3D) and the possibility myocardial deformation (Strain) analysis by the speckle tracking technique.^1-3^

Unlike two-dimensional echocardiography, where geometric inferences and mathematical calculations are required, the echo3D allows direct measurement of ventricular and atrial volumes, from which function data such as left and right ventricular ejection fraction, and left atrial function, including total emptying fraction and active emptying fraction parameters.^4-7^

In addition, left ventricular ejection fraction by echo3D proved to be a better prognostic parameter than the ejection fraction obtained by two-dimensional imaging.^8^ Furthermore, echo3D has better reproducibility and correlation with cardiac magnetic resonance than two-dimensional echocardiography.^1,9,10^

More recently, the speckle tracking technique for the measurement of myocardial deformation (Strain) has been developed. The strain is a novel parameter for the assessment of left and right ventricular and atrial contractile function.^1,3^

Myocardial strain is a parameter that seems to change before ejection fraction in several diseases that may evolve with impairment of systolic ventricular function, including cardiomyopathies, valvular heart disease, cardiotoxicity induced by chemotherapy, pulmonary hypertension, among others, and it has prognostic value in different conditions.^1,3,11-13^

It is essential to have reference values to the use of these new technologies in clinical practice. International guidelines suggest some reference values, but they are based on few studies.^1-3,6,14^ More recently, the European Association of Cardiovascular Imaging conducted a multicenter study that included 440 subjects to determine real-time three-dimensional and Strain echocardiographic reference values ​​for the European population.^6^

Thus, the study published by Saraiva et al.^15^ in this issue has great importance in the Brazilian scenario, since the reference values ​​from international publications are not always appropriate for use in the Brazilian population, which has peculiar ethnic distribution and miscegenation.^15^

The authors were concerned with selecting a group of individuals that were representative of the Brazilian population, including a population with an ethnic distribution similar to that observed in the IBGE demographic census.^15^ Also noteworthy is the assessment of sorology for Chagas in all individuals.

The reference values ​​were determined for different parameters obtained by three-dimensional echocardiography, including diastolic and systolic ventricular volumes, as well as the left ventricular ejection fraction and the different left atrial volumes throughout the cardiac cycle, allowing to determine their total, passive and active emptying fractions.^15^

Regarding the parameters derived from speckle tracking, the reference value of the global left ventricular longitudinal strain was determined - the most reliable parameter and used to evaluate myocardial deformation^1,3^ - as well as the radial strain and circumferential strain of this chamber. In addition, it was determined the right ventricular global and free wall Strain.^15^

In addition, normal values for the different atrial strains throughout the cardiac cycle and left ventricular basal and apical rotation, twist, untwist, and torsion were determined.^15^

Considering the use in clinical practice of values between two below and above average standard deviations as normal values, we can conclude that Brazilian echocardiography laboratories can already implement these new reference values for the Brazilian population when using the new echocardiographic techniques, without having to use reference values obtained in other populations.
